# The Epidemiology of Pediatric Bone and Joint Infections in Cambodia, 2007–11

**DOI:** 10.1093/tropej/fms044

**Published:** 2012-09-13

**Authors:** Nicole Stoesser, Joanna Pocock, Catrin E. Moore, Sona Soeng, PutChhat Hor, Poda Sar, Direk Limmathurotsakul, Nicholas Day, Varun Kumar, Sophy Khan, Vuthy Sar, Christopher M. Parry

**Affiliations:** ^1^Angkor Hospital for Children, Siem Reap, Cambodia; ^2^Wellcome Trust Major Overseas Program, Mahidol–Oxford Tropical Medicine Research Unit, Bangkok, Thailand; ^3^Centrer for Clinical Vaccinology and Tropical Medicine, Churchill Hospital, Oxford University, Oxford, UK; ^4^Department of Medicine, Addenbrooke’s Hospital, Cambridge, UK; ^5^University of Cambridge, Cambridge, UK

**Keywords:** osteomyelitis, septic arthritis, pediatric, Asia

## Abstract

There are limited data on osteoarticular infections from resource-limited settings in Asia. A retrospective study of patients presenting to the Angkor Hospital for Children, Cambodia, January 2007–July 2011, identified 81 cases (28% monoarticular septic arthritis, 51% single-limb osteomyelitis and 15% multisite infections). The incidence was 13.8/100 000 hospital attendances. The median age was 7.3 years, with a male/female ratio of 1.9:1; 35% presented within 5 days of symptom onset (median 7 days). *Staphylococcus aureus* was cultured in 29 (36%) cases (52% of culture-positive cases); one isolate was methicillin-resistant (MRSA). Median duration of antimicrobial treatment was 29 days (interquartile range 21–43); rates of surgical intervention were 96%, and 46% of children had sequelae, with one fatality. In this setting osteoarticular infections are relatively common with high rates of surgical intervention and sequelae. *Staphylococcus aureus* is the commonest culturable cause, but methicillin-resistant *S. aureus* is not a major problem, unlike in other Asian centers.

## Introduction

Pediatric osteomyelitis and septic arthritis are severe infections, and diagnostic delay is associated with poor outcomes [1–3]. The incidence of pediatric osteomyelitis varies from 2.9 to 75 cases/100 000 individuals, and that of septic arthritis varies from 5 to 37 [4–9]. Unlike the increasing incidence of bone infection, the incidence of joint infection appears to be stable [10–12].

Specific data on pediatric osteoarticular infections from resource-limited Asian countries, such as Cambodia, are sparse [13–19]. Risk factors such as malnutrition, trauma and suboptimal vaccine coverage are widespread [20–22]. Diagnostic microbiology facilities are limited, and antimicrobial resistance is common [23]. This study characterized the epidemiology of osteoarticular infections in Cambodian children aged <16 years attending Angkor Hospital for Children (AHC), Siem Reap.

## Material and Methods

Cases from January 2007–July 2011 were identified through hospital and laboratory records. Data collected included age, gender, residence, admission details, comorbidities, clinical presentation, laboratory investigations, surgical interventions, antibiotic treatment and outcomes. Weight-for-age *Z*-scores were calculated for children aged <5 years [24].

Disease episodes were defined as single treatment episodes, and relapses as recurrent disease episodes following improvement. Monoarticular septic arthritis described single joint infections; single-limb osteomyelitis included adjacent infected long bones within a limb and bone-plus-adjacent-joint involvement, and multisite infections included non-adjacent sites of infection, and/or non-musculoskeletal sites. Mandibular/foot infections were considered separately.

Data were analyzed using Stata 11.1 (StataCorp, TX, USA). Fisher’s exact and Kruskal–Wallis tests were used for comparisons between groups for categorical and continuous variables, respectively. Multivariable logistic regression was used to assess risk factors for binary outcomes.

Ethical approval was granted by the AHC Institutional Review Board and the Oxford Tropical Research Ethics Committee, UK.

## Results

Of 81 patients, 60 (74%) had a single episode of osteoarticular infection, and 21 had primary episodes followed by relapse(s). The median age (range) was 7.3 years (0–14); boys were almost twice as commonly afflicted. Trauma was seen in 56% of cases, 41% of which was penetrating. Where vaccination status was known, 52 (74%) children had received age-appropriate vaccinations. Five of 23 (22%) under-5 s were moderately to severely undernourished.

**Fig. 1.**
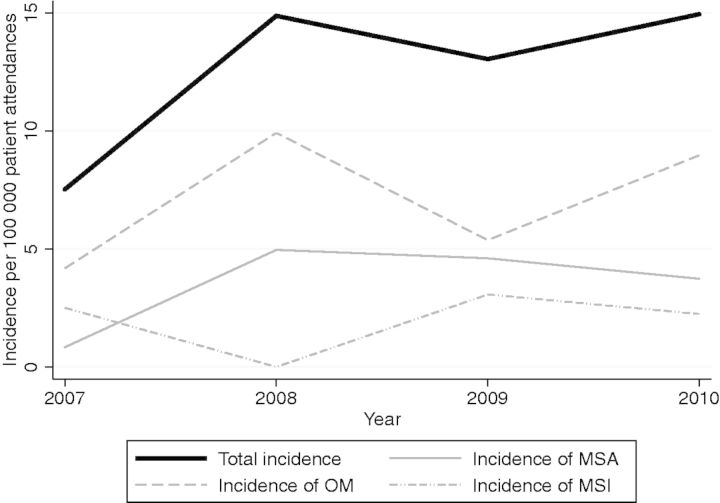
Changes in incidence by clinical syndrome, 2007–2010. MSA, monoarticular septic arthritis; OM, single-limb osteomyelitis (includes single-limb osteomyelitis with adjacent joint involvement); MSI, multisite infection. No observed changes were statistically significant.

For primary episodes, details across clinical categories and symptoms are represented in Tables 1 and 2. There were no significant differences in white cell count (WCC), ESR or CRP between clinical groups. Crude incidence for non-relapses was 13.8/100 000 attendances; temporal changes by clinical group are depicted in Fig. 1. Median follow-up time (interquartile range [IQR]; range) was 28.5 days (0–140; 0–1339).

**Table 1:** Demographic, admission and investigation results associated with clinical syndromes

	Monoarticular septic arthritis (*n* = 23)	Single-limb osteomyelitis with/without adjacent joint infection (*n* = 41)	Multisite infections (*n* = 12)	*p*-value
Median age, years (IQR)	6.7 (1.1–11.2)	6.4 (3.1–9.5)	9.25 (6.15–11.85)	0.21
Male, *n* (%)	15 (65)	29 (71)	5 (42)	0.17
Median duration of symptoms, days (IQR)	5 (2–28)	21 (6–45)	5.5 (3–6)	0.003
Positive blood culture, *n* (% of total patients sampled)	5 (56)	4 (50)	10 (91)	0.10
*Staphylococcus aureus* infection, *n* (%)	4 (17)	26 (63)	12 (100)	<0.001
Median length of stay, days (IQR)	12 (4–13)	13 (8–16)	28 (19–40)	<0.001
Number admitted to intensive care (%)	0	1 (3)	7 (58)	<0.001
Median length of stay in intensive care, days (IQR)		1	8.5 (4–10)	0.13

Cases of mandibular (*n* = 2), calcaneal (*n* = 2) and metatarsal osteomyelitis (*n* = 1) have been excluded from this analysis.

**Table 2:** Symptoms associated with particular clinical syndromes

Symptom (%)	Monoarticular septic arthritis (*n* = 22)^a^	Single-limb osteomyelitis with/without adjacent joint infection (*n* = 41)	Multisite infections (*n* = 12)	*p*-value
Fever, *n* (%)	19 (86)	30 (73)	11 (100)	0.11
Bone/joint pain, *n* (%)	21 (95)	35 (85)	10 (91)	0.60
Decreased movement, *n* (%)	18 (82)	28 (68)	9 (82)	0.50
Erythema, *n* (%)	5 (23)	21 (51)	7 (64)	0.04
Swelling, *n* (%)	14 (64)	37 (90)	11 (100)	0.009
Respiratory, *n* (%)	1 (5)	3 (7)	8 (73)	<0.001

Cases of mandibular (*n* = 2), calcaneal (*n* = 2) and metatarsal osteomyelitis (*n* = 1) have been excluded from this analysis.

^a^Clinical details not available for one case.

Many patients sought treatment before attendance at our institution: 48% had been reviewed in a private clinic and/or by traditional healers, and 24% had taken antibiotics.

### Monoarticular septic arthritis

**Fig. 2.**
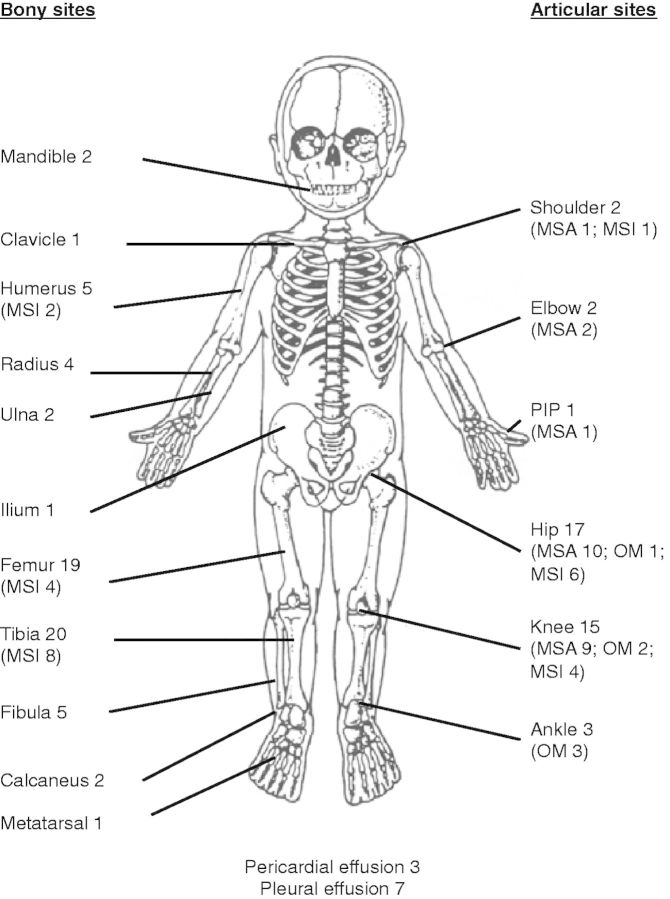
Prevalence of anatomical site involved in osteoarticular infections. MSA, monoarticular septic arthritis; OM, single-limb osteomyelitis (includes single-limb osteomyelitis with adjacent joint involvement); MSI, multisite infection; PIP, proximal interphalangeal joint.

Forty-eight percent of cases presented within 5 days of symptom onset; 68% had fever, joint pain and decreased movement. The hip was involved in 43%, and the knee in 39% of cases (Fig. 2). Microbiological results are presented in Table 3.

**Table 3:** Demographic and microbiological details for sample-positive monoarticular septic arthritis cases (n = 13)

Patient characteristics	Age (years)	Gender	Causative organism	Sites of culture positivity		Susceptibility results									
				Blood	Synovial fluid	AMP	AMC	CHL	CIP	CRO	ERY	GEN	OXA	PEN	SXT
1	1.1	F	*Haemophilus influenzae*	*H. influenzae*	Gram-negative bacilli on microscopy; no growth on culture	R	S		S	S		R			S
2	0.7	M	*H. influenzae*	Sample not taken	*H. influenzae*	S	S		S	S		S			R
3	14.2	M	*Streptococcus pyogenes*	Gram-positive cocci on microscopy; no growth on culture	*S. pyogenes*					S	S			S	
4	9.9	F	Beta-hemolytic streptococcus^a^	Beta-hemolytic streptococcus	Negative					S	S			S	
5	9.1	F	*Salmonella enterica* Typhi	*S.* Typhi	Negative	R		R	R^b^	S					R
6	11.8	F	*Staphylococcus aureus*	*S. aureus*	Gram-positive cocci on microscopy; no growth on culture				S		R	S	S^c^	R	S
7	7.8	M	*S. aureus*	Negative	*S. aureus*				S		S	S	S	R	S
8	6.7	F	*S. aureus*	Negative	*S. aureus*				S		S	S	S	R	S
9	13.1	F	*S. aureus*	Not done	*S. aureus*				S		R	S	S	R	S
10	0.8	M	Unspecified gram-negative bacillus	Not done	Gram-negative bacilli on microscopy; no growth on culture		Susceptibility testing not done								
11	1.8	M	Mixed growth	Not done	No Gram stain results; Mixed growth of *Enterobacter aerogenes*, *Streptococcus pneumoniae* and an unspeciated gram-negative bacillus		Susceptibility testing not done								
12	9.1	M	Unspecified gram-positive bacillus		Gram-positive bacilli on microscopy; no growth on culture		Susceptibility testing not done								
13	14	F	Unspecified Gram-positive bacillus		Gram-positive bacillus on microscopy of two aspirates; growth of unidentified Gram-positive bacillus on culture		Susceptibility testing not done								

Antimicrobial abbreviations as follows: AMP, ampicillin; AMC, co-amoxiclav; CHL, chloramphenicol; CIP, ciprofloxacin; CRO, ceftriaxone; ERY, erythromycin; GEN, gentamicin; OXA, oxacillin; PEN, penicillin; SXT, co-trimoxazole.

^a^Lancefield grouping not done.

^b^Reduced susceptibility to ciprofloxacin.

^c^Methicillin susceptibility inferred from oxacillin susceptibility.

Aspiration/drainage was performed in 21 of 22 cases, with 55% having multiple aspirations; five cases required subsequent arthrotomies. Six had arthrotomies without preceding aspiration. The median (IQR) duration of antimicrobial treatment was 27 days (10–29), with 10 days of intravenous treatment. Neither hip joint involvement nor arthrotomy was associated with sequelae (*p* = 0.66, 1.0).

### Single-limb osteomyelitis

On presentation, the median (IQR) duration of symptoms was 17.5 days (6–60). Forty-six percent presented acutely (<14 days of symptoms), 37% subacutely (≥14 ≤90) and 17% with chronic osteomyelitis (>90). The femur (37% of cases) was most commonly affected (Fig. 2).

A higher mean admission WCC was associated with acute presentations (21.1 vs. 11.7 × 10^9^/l; *p* < 0.001). Mean values for WCC, ESR and CRP were non-significantly higher in those with sequelae.

Fifty percent of admission blood cultures were positive. Bacterial pathogens were cultured from 25 bone/pus samples from 37 individuals: 22 with *Staphylococcus aureus* alone (susceptibilities in Table 4), one with *Haemophilus influenzae* and two with mixed infections (one *S. aureus*/*Escherichia coli*; one *S. aureus*/beta-hemolytic streptococcus). No methicillin-resistant *S. aureus* (MRSA) was isolated. One case was positive for acid-fast bacilli on microscopy.

**Table 4:** Antimicrobial susceptibilities of S. aureus isolates in osteomyelitis cases (n = 24)

Susceptibility pattern	Number of cases (%)
Fully susceptible to first line antibiotics^a^	1 (4)
Resistant to penicillin	9 (38)
Resistant to penicillin + erythromycin	6 (25)
Resistant to penicillin + co-trimoxazole	5 (21)
Resistant to penicillin + erythromycin + co-trimoxazole	2 (8)
Resistant to erythromycin	1 (4)

^a^Defined as penicillin, erythromycin, gentamicin, ciprofloxacin, trimethoprim, oxacillin.

All but two patients had surgery. The median duration of antimicrobial therapy (IQR) was 30 days (25–43) with 12 days of intravenous therapy.

Both mandibular cases were associated with poor dentition; one had health care-associated MRSA osteomyelitis. The three cases with foot osteomyelitis all occurred post-puncture wounds.

### Multisite infections

Multisite infections were invariably associated with *S. aureus*. They included most cases (7/8) admitted to intensive care and the only fatality.

### Sequelae

Excluding foot and mandibular cases, 35 (46%) children had sequelae (Table 5), of which 20 were relapses. The only risk factor for sequelae was the use of antimicrobial therapy for >30 days [adjusted odds ratio 6.4 (95% CI 1.6–25.5), *p* = 0.008].

**Table 5:** Summary of relapse and outcome data for patients with particular clinical syndromes

Outcome	Number of patients by clinical group		
	Monoarticular septic arthritis	Single-limb osteomyelitis ± adjacent joint infection	Multisite infections
Outcomes at final follow-up in patients without relapse (*n* = 56)			
Decreased movement (%)	3 (13)	7 (24)	2 (40)
Residual pain (%)	0	1 (3)	0
Persistent fever (%)	1 (5)	0	0
Fever and decreased movement (%)	1 (5)	0	0
Death (%)	0	0	1 (20)
Considered cured (%)	17 (77)	21 (72)	2 (40)
Outcomes at final follow-up in patients with at least one relapse (*n* = 20)			
Decreased movement	0	4 [+1]^a^ (38)	2 (33)
Limb swelling	0	2 [+1]^a^ (24)	0
Considered cured	1 (100)	5 (38)	4 (67)

Cases of mandibular (*n* = 2), calcaneal (*n* = 2) and metatarsal osteomyelitis (*n* = 1) have been excluded from this analysis.

^a^Numbers in square brackets denote status at last visit; however, further follow-up was planned for these two patients beyond the study end period.

Of the cases known to have relapsed, 15 relapsed once and five had multiple relapses. Final outcomes are shown in Table 5.

## Discussion

This study is one of the larger, recent, single-center series of pediatric osteoarticular infection, and the only study, to our knowledge, from Cambodia. Interstudy comparisons of incidence are difficult, as denominators are frequently different [5–7, 25]; however, osteoarticular infections represent a relatively common surgical problem locally, accounting for approximately 1 in 50 surgical admissions.

*Staphylococcus aureus* was the most commonly cultured pathogen, but MRSA was not a major problem, unlike in other regional studies [13, 14]. Six percent of cases cultured *H. influenzae*, demonstrating the importance of empirical cover for this in the absence of adequate vaccination.

More than 95% of patients required surgical intervention, and rates of infectious sequelae were high, although we did not find any associations with published risk factors for poor outcomes [15, 26]. Delays in presentation and inappropriate preliminary treatment are likely to be contributing factors. A further concern is the use of low-dose/low-frequency oral antibiotic regimens in treatment to improve compliance. Studies of suitable regimens in our setting are needed.

Study limitations include incomplete follow-up; this and the sample size have made it unfeasible to model risk factors for relapse. Diverse treatment approaches were used, making it difficult to identify optimal regimens. We did not look for *Kingella kingae*, which is an important pathogen elsewhere [27, 28]. Nevertheless, we demonstrate relevant features of these infections in Cambodian children, which can be used as a baseline for modifications to treatment approaches and the monitoring of epidemiological trends.

## Funding

This work was supported by the Wellcome Trust of Great Britain and the Li Ka Shing–University of Oxford Global Health Program.
